# Toxicity of a Binary Mixture of TiO_2_ and Imidacloprid Applied to *Chlorella vulgaris*

**DOI:** 10.3390/ijerph18157785

**Published:** 2021-07-22

**Authors:** Cristina Adochite, Luminita Andronic

**Affiliations:** Product Design, Mechatronics and Environment Department, Transilvania University of Brasov, Eroilor 29, 500036 Brasov, Romania; cristina.adochite@unitbv.ro

**Keywords:** toxicity, TiO_2_ Degussa, imidacloprid, *Chlorella vulgaris*, photocatalysis

## Abstract

Nanoparticles have applications in various fields such as manufacturing and materials synthesis, the environment, electronics, energy harvesting, and medicine. Besides many applications of nanoparticles, further research is required for toxic environmental effect investigation. The toxic effect of titanium dioxide nanoparticles on the physiology of the green alga *Chlorella vulgaris* was studied with a widely used pesticide, imidacloprid (IMD). *Chlorella vulgaris* was exposed for 120 h in Bold’s basal medium to different toxic compounds, such as (i) a high concentration of TiO_2_ nanoparticles, 150–2000 mg/L, usually optimised in the photocatalytic degradation of wastewater, (ii) an extremely toxic pesticide for the aquatic environment, imidacloprid, in concentrations ranging from 5 to 40 mg/L, (iii) TiO_2_ nanoparticles combined with imidacloprid, usually used in a photocatalytic system. The results show that the TiO_2_ nanoparticles and IMD inhibited *Chlorella vulgaris* cell growth and decreased the biovolume by approximately 80% when 2 g/L TiO_2_ was used, meaning that the cells devised a mechanism to cope with a potentially stressful situation; 120 h of *Chlorella vulgaris* exposure to 40 mg/L of IMD resulted in a 16% decreased cell diameter and a 41% decrease in cell volume relative to the control sample, associated with the toxic effect of pesticides on the cells. Our study confirms the toxicity of nanoparticles through algal growth inhibition with an effective concentration (EC50) value measured after 72 h of 388.14 mg/L for TiO_2_ and 13 mg/L for IMD in a single-toxic system. The EC50 of TiO_2_ slowly decreased from 258.42 to 311.11 mg/L when IMD from 5 to 20 mg/L was added to the binary-toxic system. The concentration of TiO_2_ in the binary-toxic system did not change the EC50 for IMD, and its value was 0.019 g/L. The photodegradation process of imidacloprid (range of 5–40 mg/L) was also investigated in the algal medium incubated with 150–600 mg/L of titanium dioxide.

## 1. Introduction

Nanoparticles have applications in various fields such as energy, chemistry, materials and environmental testing, electronics and communications, automotive, medicine, and cosmetics. These various applications have led to an intensification of the synthesis of these nanomaterials in the last decade, which makes it necessary to test them for their toxicity in the aquatic environment.

Titanium dioxide is widely used in electronics and automotive industries, has applications in environmental remediation, and has been investigated for over 40 years in solar cell development [1]. In water purification processes, titanium dioxide is used to degrade organic pollutants, especially pesticides [1], dyes [2,3], and pharmaceuticals [4]. Pesticide water contamination is mainly caused by intensive irrigation, pesticide disposal as a standard agricultural practice, and rinsing pesticide-polluted water from tanks, spray equipment, and agricultural wastewater (fruit and vegetable washing or postharvest treatment) [5].

One pesticide that is widely used in agriculture is imidacloprid (IMD). Imidacloprid is highly soluble in water (0.58 g/L) [6] and has a long half-life of more than 33–44 days in water [5,7]. Several groups around the world have extensively studied the photodegradation of TiO_2_-assisted imidacloprid. [8,9,10,11,12,13,14]. In addition, imidacloprid has a large geographical distribution and sensitivity to toxicants [14].

The introduction of nanomaterials and pesticides into aquatic environments resulting from excessive use has prompted researchers to correlate the properties, origins, behaviours, and toxicological consequences. When nanoparticles (NPs) and pesticides are released into the aquatic world, they can undergo various transition processes, determining how they affect biological systems. The properties of nanoparticles, such as morphology, grain size, surface area, crystallinity, and chemistry, influence the toxicity of the organisms present in the aquatic environment. Green algae play an important role in the aquatic ecosystem, so they can also be used as a model to assess the toxic effects of nanoparticles used in water purification processes [15]. Growth medium (nutrients), light (type, duration and intensity), temperature, pH, conductivity, and dissolved oxygen all have a role in microalgal growth, e.g., *Chlorella vulgaris*. These factors are frequently regulated in a laboratory setting; however, there is far greater variance in irradiance and day length in the environment. In microalgal mass culture technology, optimising output is critical, and day length is a determining element in microalgae development. The circadian rhythm of photosynthesis, respiration, cell division, and growth rates is influenced by the length of the day [16]. Other algal parts and components, such as unsaturated fatty acids, carbohydrates, and protein, can be influenced by the light regime in addition to pigment concentration [17]. Nanomaterials are easily transferable to aquatic environments. While nanomaterials have various advantages, their total toxicity is still unknown. The few research studies on the hazardous effects of nanoparticles on natural systems are inconsistent, if not contradictory [18]. The high abundance of toxins in aquatic environments targets microalgae, which are key primary producers [19]. Because they are the initial level of the trophic web, any problems they confront will eventually impact the rest of the ecosystem [20]. Because of their high sensitivity to manufactured nanoparticles and their capacity to accumulate them, marine algae can be utilised as a pollutant indicator in marine environments [21]. The algae have a high vulnerability to nanomaterials due to low 50 per cent effective concentrations (EC50) [22]. Many metallic oxides such as SiO_2_ [23], Al_2_O_3_ [24], TiO_2_ [25], and ZnO [26] are used to study the toxic effects on algae. At the NP-dependent concentration and due to their intrinsic properties, they are known to cause growth inhibition and further algal death [25].

To our knowledge, no one has investigated the effect of a binary system of TiO_2_-imidacloprid on the evolution of the green alga, *Chlorella vulgaris*, correlated with photocatalysis processes. In this paper, we investigate the toxic effect of TiO_2_ nanoparticles and pesticides, particularly imidacloprid, on the growth of *Chlorella vulgaris*. Effective concentrations (EC50) were experimentally determined after 72, 96, and 120 h to quantify the toxic effect of TiO_2_ and imidacloprid (IMD, TiO_2_ concentration needed to induce a 50% reduction in growth).

## 2. Materials and Methods

### 2.1. Algal Culture

*Chlorella vulgaris* (CCAP211/11B) was supplied by the Algae and Protozoa Culture Collection (CCAP) of the Scottish Marine Institute (Dunberg, Scotland).

A standard Bold Basal medium (BBM) contained all nutrients: NaNO_3_ (25 g/L), CaCl_2_∙2H_2_O (2.5 g/L), MgSO_4_·7H_2_O (7.5 g/L), K_2_HPO_4_∙3H_2_O (7.5 g/L), KH_2_PO_4_ (17.5 g/L), NaCl (2.5 g/L), and traces elements were used in the growth experiments. A trace element solution based on 0.75 g Na_2_EDTA, 0.097 g FeCl_3_∙6H_2_O, 0.041 g MnCl_2_∙4H_2_O, 0.005 g ZnCl_2_, 0.002 g CoCl_2_∙6H_2_O, and 0.004 g Na_2_MoO_4_∙2H_2_O relative to a 1 L solution was used to complete the need for nutrients. One litre of BB medium contains the following volumes of the solutions listed above: 30 mL NaNO_3_, 10 mL of each solution of CaCl_2_, MgSO_4_, K_2_HPO_4_, KH_2_PO_4_, NaCl, and 6 mL of trace element solution.

*Chlorella vulgaris* with an initial concentration of approximately 500 × 10^4^ cells/mL was maintained in 250 mL of fresh BBM at a temperature around 25.0 °C under a 16 h:8 h light: dark controlled by a timer. The cultures were irradiated with five tubes of fluorescent F18/T8 Sylvania aquastar (18 W, 900 lux each). The photosynthetically active radiation (PAR) was approximately 60–70 μmol·m^−2^·s^−1^, measured with the quantum sensor LI-190R-BNC-2 attached to the LI-250A light meter (LI−COR Inc., Lincoln, NE, USA). In addition, conductivity, dissolved oxygen and pH were measured daily using a multiparameter pH-meter Edge HI2020 (Hanna Instrument) (Table S1).

### 2.2. Toxicity of TiO_2_ and IMD as Individual and Binary Mixtures

**Figure 1:**
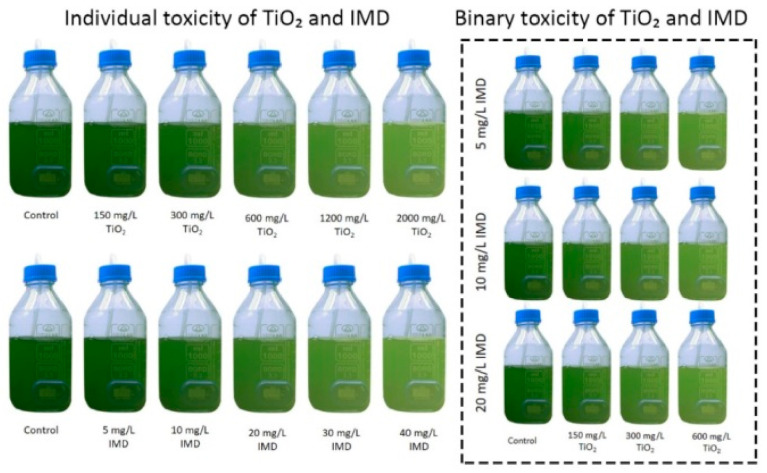
The experimental description of individual and binary toxicity of TiO_2_ nanoparticles and IMD pollutant.

Toxicity of TiO_2_ and IMD as individual and binary mixtures towards *Chlorella vulgaris* was investigated at various toxic concentrations such as 150–2000 mg/L for TiO_2_ and 5–40 mg/L for IMD. The schematic representation of the experiments conducted for the toxicity tests is seen in Figure 1. The conditions were maintained continuously during 120 h of the experiments, and the cultures were not fed during the test period.

According to ISO 8692/2012, the pH of the algal culture was adjusted with 1 M NaOH concentration solution to 8.1. At the end of the incubation period (120 h), pH, conductivity, and dissolved oxygen were recorded to control interference with the final results (Tables S2–S4). The values indicate minor changes in the parameters investigated, pH, conductivity or dissolved oxygen, under experimental conditions. The pH increased from 8.1 to 9 in the control after 120 h, while the pH decreased slightly with increasing TiO_2_ concentration, IMD concentration, and mixture. Conductivity and dissolved oxygen were retained with respect to the initial parameters.

### 2.3. Algal Growth Analysis

The determination of the growth of the green algal culture was done by determining the cell concentration up to 120 h; the measurement was made daily. The cell concentration was determined using an Improved Neubauer cytometer (Marienfeld), following the procedure: (i) the cytometer and the coverslip were cleaned with ethanol and dried, (ii) the coverslip was placed over the grid on the cytometer, (iii) a 10 μL volume of the suspension containing the algae was placed at the edge of the coverslip, (iv) the number of cells in five separate squares was recorded, and the concentration of cells was calculated with Equation (1) according to a standardised protocol in our laboratory; any cell found on the left and top line was quantified, and any cells found on the right and bottom lines of each square were ignored. All measurements were performed in duplicate (Equation (1)).

(1) $$Cell\;Number\;\left(\frac{cells}{mL}\right)=\frac{Total\;number\;of\;cells\;counted}{Number\;of\;squares\;counted}\cdot 10^{4}$$

In experiments performed for toxicity tests, the direct measurement of cells by this method, although time-consuming, has several advantages such as any microscopic abnormality of the cells is observed under the optical microscope, and the nanoparticles present with the cells do not interfere with the measurement, as is the case with optical density or biomass.

The initial cell density was kept around 500 × 10^4^ cells/mL, and they were taken in the exponential growth process. The experiments were carried out for five days.

The growth rate of batch cultures (μ) expressed as day-1 was calculated daily using Equation (2) [27]. Growth was measured as the increase in cell density over the test period (Equation (2)).

(2) $$\mu =\frac{lnN_{t}-lnN_{0}}{t_{}}$$

where *N*_0_ is initial cell density at time 0 (cells/mL), Nt is the cell density at time *t* (cells/mL), *t* = time (day).

The diameter of the algae was determined by visualising 15 cells under an optical microscope and randomly choosing diameter cells using the software MikroCamLabII. First, the cell numbers (N) were counted every 24 h during the exposure time (120 h) using an optical microscope with a lens 40× (Bresser, Rhede, Germany). Then, using diameter data (d, μm), the mean volume per cell (V, μm3) was calculated (Equation (3)). Furthermore, the biovolumes of the cells (the volume occupied by cells in a sample) were calculated with Equation (4), assuming a spherical shape for algae [28] (Equations (3) and (4)).

(3) $$V\;\left(\mu m^{3}\right)=\frac{\pi }{6}d^{3}$$

(4) $$Biovolume\;\left(\mu m^{3}\right)=V\cdot N$$

The biovolume represents potential trade-offs between important physiological characteristics, such as cell volume and density (Romero et al., 2020). On the other hand, the biovolume proved to be an insensitive parameter in determining the toxicity of nanoparticles and pesticide to *Chlorella vulgaris*.

Microalgal development was also monitored daily using a UV-VIS spectrophotometer to detect optical density (OD) at wavelength 680 nm and 750 nm for turbidity value (OD680, OD750) (Lambda 25, Perkin Elmer). The relationship between OD680 and OD750 for *Chlorella vulgaris* was previously established by a linear curve. Biomass concentration (expressed as dry weight concentrations in mg/L) was also determined to assess the rate of algal growth and development using 100 mL of filtered culture using a vacuum pump on a 47 mm glass microfiber filter (Branchia). The filter was dried and weighed to give the exact mass of only the microalgal cells. The Lichtenthaler method (Lichtenthaler, 1987) was used to determine photosynthetic pigment production with minimal adjustments. A 10 mL culture was centrifuged at 4000 rpm for 5 min, the supernatant was discarded, and the pellet was resuspended in 10 mL of 90% methanol. After that, the sample was sonicated for five minutes, incubated, and centrifuged (4000 rpm, 5 min). The supernatant was collected, and the process was repeated until the cells turned white (all pigments were extracted). The collected supernatant was analysed spectrophotometrically on a spectrum of 200–800 nm. The photosynthetic pigments, chlorophyll a, chlorophyll b, total chlorophyll, and total carotenoid content, were evaluated by spectrophotometric measurements at wavelengths 665 nm (A665), 652 nm (A652), and 468 nm (A468) nm, and turbidity corrections (A750) were made using the following Equations (5)–(8).

(5) $$Chlorophyll\;a\;\left(\frac{\mu g}{mL}\right)=\frac{\left[16.72\cdot \left(A_{665}-A_{750}\right)-9.16\cdot \left(A_{652}-A_{750}\right)\right]\cdot 15}{1\cdot 10}$$

(6) $$Chlorophyll\;b\;\left(\frac{\mu g}{mL}\right)=\frac{\left[34.09\cdot \left(A_{652}-A_{750}\right)-15.28\cdot \left(A_{665}-A_{750}\right)\right]\cdot 15}{1\cdot 10}$$

(7) $$Total\;chlorophyll\;\left(\frac{\mu g}{mL}\right)=\frac{[(-1.44\cdot \left(A_{665}-A_{750}\right)+24.93\cdot \left(A_{652}-A_{750}\right)\cdot 15}{1\cdot 10}$$

(8) $$Carotenoids\;\left(\frac{\mu g}{mL}\right)=\frac{\left[1000\cdot \left(A_{468}-A_{750}\right)-1.63\cdot Chl\;a-104.96\cdot Chl\;b]\;\right)}{221}\cdot \;\frac{15}{1\cdot 10}$$

The amount of plant pigment in a product is an essential quality indicator of the growth rate. In addition, plant physiological, biochemical, and genetic characteristics and environmental conditions such as light, temperature, and pH influence the accumulation of chlorophylls and carotenoids [29].

The median effective concentration (EC50) is a statistical calculation of a toxicant’s concentration expected to produce the death of 50% of *Chlorella vulgaris* cells. The EC50 is often used in ecotoxicology to indicate the toxicity of a compound to the environment. The dose–response point was assessed after 72, 96, and 120 h, and EC20 and EC50 were obtained from the dose–response regression curve by plotting the logarithmic value of concentrations against the specific growth rate.

During the 120 h, the IMD concentration was evaluated daily by high-pressure liquid chromatography (HPLC) to monitor the possibility of photodegradation of the pesticide in the algae lample in the presence of titanium dioxide [5]. HPLC analysed aqueous solutions of imidacloprid, consisting of a Shimadzu LC-20ADsp chromatograph, equipped with a Nucleosil C18- Macherey Nagel, Nucleosil^®^ 5 µm C18 100 A, LC Column 250 × 4.6 mm, and a UV detector SPD-20A at an absorption wavelength of 270 nm. The column was maintained at 40 °C. The mobile phase was a mixture of 70% v acetonitrile and 30% v ultrapure water—the flow rate was 1.2 mL/min with 10 μL sample injection volume. The retention time was 2.76 min.

## 3. Results

### 3.1. Growth of Chlorella vulgaris in Batch Culture

**Figure 2:**
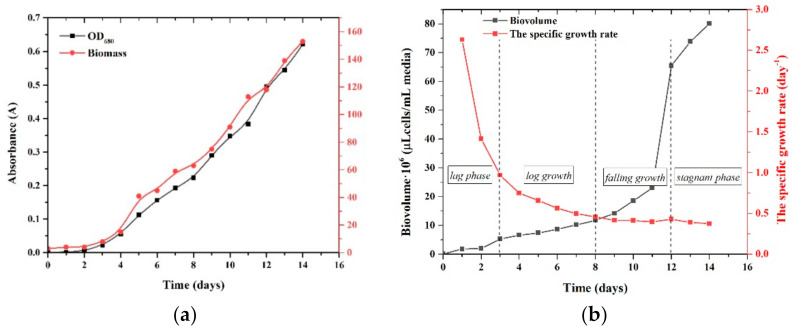
*Chlorella vulgaris* culture growth curves depending on absorbance at 680 nm and biomass concentration (**a**) and by biovolume and specific growth rate (**b**).

This test was performed to determine the tendency of cell growth. Figure 2 shows the development of algae over 14 days. The monitoring of the cellular development of *Chlorella vulgaris* was performed by measuring the OD at 680 nm and biomass concentration (Figure 2a) and by determining the specific growth rate (day-1) according to cell concentration using an optical microscope and by biovolume (Figure 2b). These results suggest that there was no adaptation period for any of the assays and that the exponential growth phase lasted about 14 days. Furthermore, no cell loss was seen during the 14-day batch culture, indicating that the trials might be continued (Figure 2). Microalgal growth characteristics, such as the specific growth rate, biomass, and biovolume, were determined and shown in Figure 2 to supplement the analysis from growth curves.

Five distinct phases of algal growth have been identified [30]: lag or acclimation, log growth, falling growth, stagnant, and lysis. The growth curve roughly follows this normal development curve with BBM growth medium composition. According to Figure 2, the lag phase lasts between 0 and 3 days, and the exponential phase lasts between 4 and 12 days. Thus, the nutrient composition takes 12 days to cycle through the typical growth phases at the recommended medium composition (BBM). When the normal algal growth curve is applied to the growth media composition, it can be seen that at the 12 day mark, the particular algal culture had only finished the falling growth phase (between 8–12 days) and had yet to reach the stationary and lysis phases. On day 12 of algal development, a sudden increase in culture was observed, given the values of biovolume occupied by cells (Figure 2b). Throughout the 14-day experiment, particular attention was paid to pH variations (8.1–10.61). Therefore, the relationship between growth and pH must be analysed (Table S1) [31].

**Figure 3:**
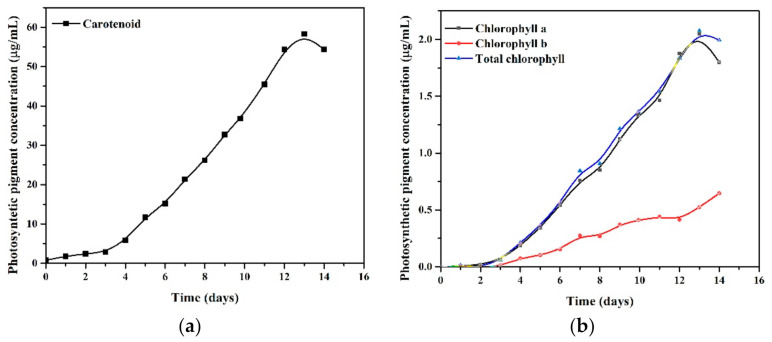
Photosynthetic pigment production (μg/mL): carotenoids (**a**) and chlorophyll-a, chlorophyll b, total chlorophyll, respectively (**b**), and throughout the 14-day experimental period (algal growth without contamination).

The chlorophyll-a concentration increased exponentially by 2.05 mg/mL in 13 days, higher than chlorophyll b, showing only a maximum value of 0.64 mg/mL after 14 days. A significant increase in carotenoid concentration, approximately 58.27 mg/mL in 13 days of growth development (Figure 3a), can be observed compared to pigments (chlorophyll a and b) (Figure 3b). According to Zhang [32], the pH of the cultures improves as carbon and nitrogen sources are assimilated, which is associated with growth and photosynthetic pigment synthesis. This could explain the values with decreased pigments (carotenoids, chlorophyll a, and total chlorophyll) on day 14, given in Figure 3. In contexts of the industrial importance of photosynthetic pigments, carotenoids are widely used in food, cosmetic products, and health, and the simultaneous extraction of valuable biological pigments, such as carotenoids, for biofuel production has been a target of research in order to make microalgal biomass even more environmentally sustainable and feasible [33].

### 3.2. Growth of Chlorella vulgaris in Batch Culture under TiO_2_ Nanoparticle Stress

The variation of cell density and biovolume over time under the stress of nanoparticles was evaluated. Biovolume is not a frequently used endpoint in toxicology assays but is the most critical algal parameter characterising water quality. Moreover, biovolume shows possible trade-offs between significant cell volume and density. For example, the biovolume of *Chlorella vulgaris* in the presence of increased concentrations of TiO_2_ and IMD showed significant changes, attributed to a decrease in cell density by the stress effect of toxicants.

**Figure 4:**
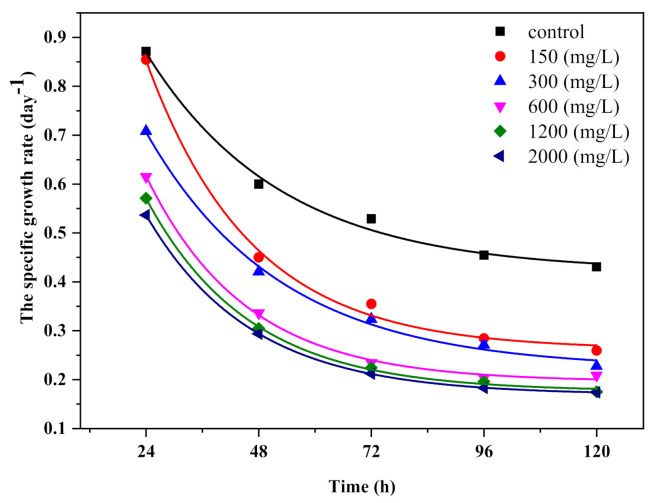
Growth curves of *Chlorella vulgaris* with different concentrations of the TiO_2_ photocatalyst.

The inhibitory effect of TiO_2_ nanoparticles on *Chlorella vulgaris* growth is shown in Figure 4. The specific growth rate decreased exponentially in time with the increase in the concentration of TiO_2_ nanomaterial to stop the growth with increasing incubation time. After 24 h of algal exposure with TiO_2_, the specific growth rate decreased for each condition tested by 85%, 71%, 62%, 57%, and 54%. The results indicate that development of *Chlorella vulgaris* was inhibited by the TiO_2_-P25/20 nanomaterial having an EC50 value after 72 h of 388.14 mg/L and an EC20 value after 72 h of 529.67 mg/L.

Significant effects of nanoparticle accumulation on microalgae seem to have been detected. The probability of meeting/interaction between titanium dioxide and *Chlorella vulgaris* was influenced by concentrations, which are described as particles per volume (Figure 4). Since microalgae expand exponentially, the ratio of microalgal cells to nanoparticles can fluctuate during the incubation period. As a result, the degree of interactions between microalgae and particles can be determined by their concentrations (assuming all microalgal and particle sizes were fixed), collision shape, and interactive surface charges titanium dioxide and microalgae in terms of concentration [34].

The decrease in photosynthetic activities will indirectly affect cell density, with a 38% reduction as calculated [15]. The average volume and biovolume occupied by the cells were calculated according to each sample’s cell diameter each day.

**Figure 5:**
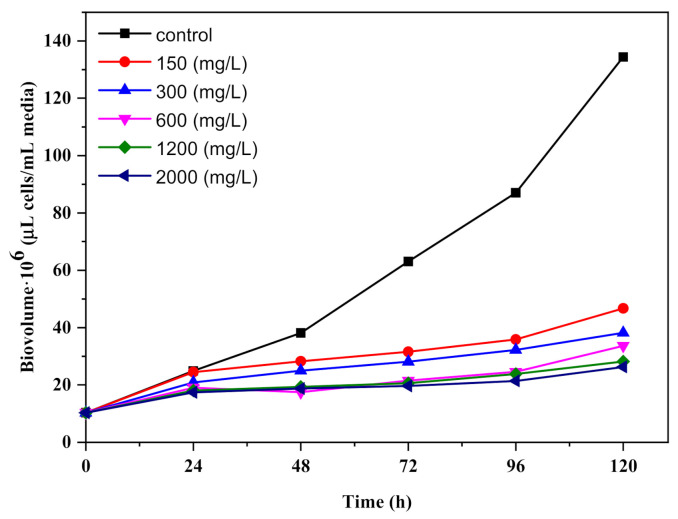
Time-dependent results of biovolume from a batch culture of *Chlorella vulgaris* under toxic TiO_2_ stress.

*Chlorella vulgaris* exposure to TiO_2_-P25/20 caused morphological changes in the cells dependent on the nanoparticle concentration and exposure time. Romero et al. showed that silver nanoparticles influenced certain morphological features of algae in a concentration- and time-dependent manner [28]. At a high concentration of TiO_2_ (2000 mg/L), there was a decrease of 11% in cell diameter and 29% in cell volume compared to the control after 120 min. The increase in the concentration of nanoparticles led to a significant increase in their toxicity to algae. The inhibitory rate in the biovolume was approximately 80%, while the concentration of TiO_2_ in the cultures was 2000 mg/L (Figure 5). These results were associated with the growth rate, implying that the cells devised a mechanism to cope with a potentially stressful situation.

Table 1 shows the different EC50s that accentuate the toxic effect of titanium dioxide nanoparticles on the different species tested. For example, the EC50 of TiO_2_ on *Chlorella vulgaris* was 293.48 mg/L after 120 h, nearly two times higher than in other studies under incubation with TiO_2_ and *Phaeodactylum tricornutum* calculated at 120 h, EC50 = 167.71 mg/L [35]. One potential explanation is that TiO_2_ toxicity was dependent on various factors, including the method used to prepare the nanoparticle suspension, size, morphology, medium, and time of exposure and the state of aggregation [36,37].

When exposed to light, some nanomaterials, such as TiO_2_, can release excited electrons, particularly when exposed to UV light [43]. Algal cells are known to require light to sustain their growth. As a result, when algal cells are exposed to nanomaterial and light, the nanoparticle ecotoxicity is clearly expressed. Photosensitivity in our study is often pointed to as the primary source of TiO_2_-P25/20 (Degussa) ecotoxicity. However, it is challenging to compare EC50s because different concentrations of nanoparticles, usually of the µg/L order, and different algae species, are usually reported.

### 3.3. Effect of the Imidacloprid on Algal Growth and Cellular Diameter

Tisler et al. described chronic toxic effects of imidacloprid on *Desmodesmus subspicatus* [44]. There are few statistics available, but they suggest low levels of imidacloprid in the environment; the lowest and highest observed environmental concentrations of imidacloprid were 1 μg/L and 14 μg/L, respectively [45]. As *Chlorella vulgaris* is exposed to IMD, it causes morphological changes in the cells dependent on the nanoparticle concentration and exposure time. The decrease in photosynthetic activities will indirectly affect cell density; the average volume and biovolume occupied by the cells were calculated according to each sample’s cell diameter each day. IMD exposure to *Chlorella vulgaris* algae caused morphological changes in the cells dependent on the pesticide concentration and exposure time. At a high concentration of IMD (40 mg/L), there was a decrease of 11% in cell diameter and 29% in cell volume compared to the control after 120 min. These effects were correlated with the cell growth rate, suggesting that the cells developed a coping mechanism to deal with a potentially stressful situation.

**Figure 6:**
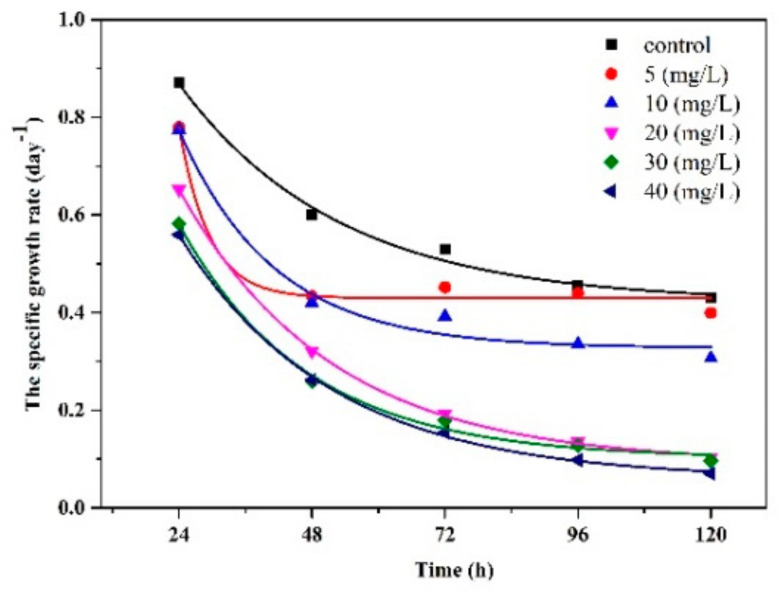
Growth curves of *Chlorella vulgaris* with different concentrations of IMD.

The inhibitory effect of imidacloprid (IMD) on *Chlorella vulgaris* is shown in Figure 6. After 24 h of exposure, the specific growth rate decreased for each condition tested by 87%, 78%, 77%, 65%, 58%, and 56%. The specific growth rate decreased in direct proportion to the increase in the concentration of IMD pesticide, which tended to stop the growth with increasing incubation time. The results indicate the development of *Chlorella vulgaris* inhibited by the IMD pesticide had an EC50 value after 72 h of 13 mg/L and an EC20 value after 72 h of 17.27 mg/L. These values decreased after 120 h, around 12 mg/L for EC50 and 16 mg/L for EC20, accentuating the toxic effect over time. Imidacloprid is currently not actively regulated in marine ecosystems.

**Figure 7:**
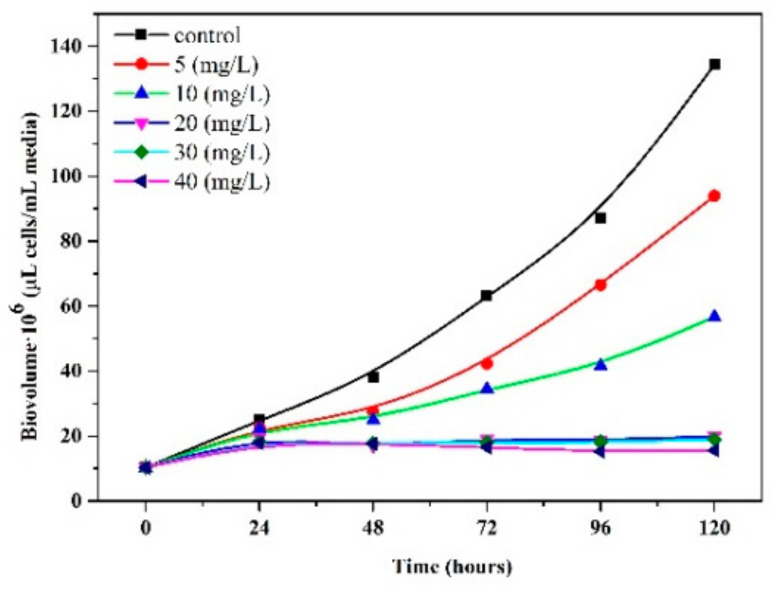
Time-dependent results of biovolume from a batch culture of *Chlorella vulgaris* under toxic IMD stress.

After 120 h of IMD (40 mg/L) stress action on *Chlorella vulgaris*, there was a 16% decrease in cell diameter and a 41% decrease in cell volume relative to the control. If the concentration of nanoparticles increased, the impact of increasing pesticide concentrations in algae cultures became more drastic. The inhibitory rate in biovolume was roughly 90%, while the IMD concentration in the cultures was 40 times greater than that in the control sample (Figure 7). These results were negatively associated with the growth rate, implying that the cells devised a mechanism to cope with a potentially stressful situation, considering the solubility property of imidacloprid. To our knowledge, two studies have also performed IMD toxicity tests. Higher toxicity of imidacloprid of EC50 = 389 mg/L after 72 h was observed by Tišler [44] for the species *Desmodesmus subspicatus*, and Malev [46] determined a value of 255 mg/L for the species *Desmodesmus subspicatus*.

### 3.4. Synergy between TiO_2_ Nanoparticles and Imidacloprid on Algae Growth

After evaluating the effects of individual exposure, the toxic effects of binary mixtures (TiO_2_ and imidacloprid in different concentrations) on the activity of algal growth were assessed. There has not been enough research on the overall toxicity of various contaminant processes. Organisms in nature are subject to a single form of pollutant and are often affected by a combination of two or more. However, the green algal exposure to the multipollutant environment and the evaluation of the contribution of each pollutant to toxicity impact are complex [47]. In addition, nanomaterials, such as titanium dioxide (TiO_2_-P25/20, Degussa), under radiation, generate reactive oxygen species (ROS) including superoxide (O_2_^•−^), hydroxyl radicals (OH^•^), and hydrogen peroxide (H_2_O_2_) [48], and, following oxidative membrane damage (cell viability), interfere with the normal function of photosynthetic organisms; as a consequence, it generates a secondary toxic impact on green algae [34]. The ROS are generated on the surface of titanium oxide nanoparticles during photocatalysis processes, disrupting cell function by lipid peroxidation, oxidising proteins, and damaging nucleic acid [49]. The generation of intracellular ROS and oxidative stress induced by NPs is frequently determined as a main toxic mechanism in the aquatic environment, particularly *Chlorella vulgaris* [50]. On the other hand, the main imidacloprid photodegradation by-products are hydroxy compounds such as 5-hydroxy imidacloprid and 4-hydroxy imidacloprid that contribute to the toxic impact on green algae [51].

The toxic binary system (IMD and TiO_2_) exposure to *Chlorella vulgaris* caused morphological changes in the cells dependent on the pesticide concentration and exposure time and the biovolume decrease (Figures S1–S3).

**Figure 8:**
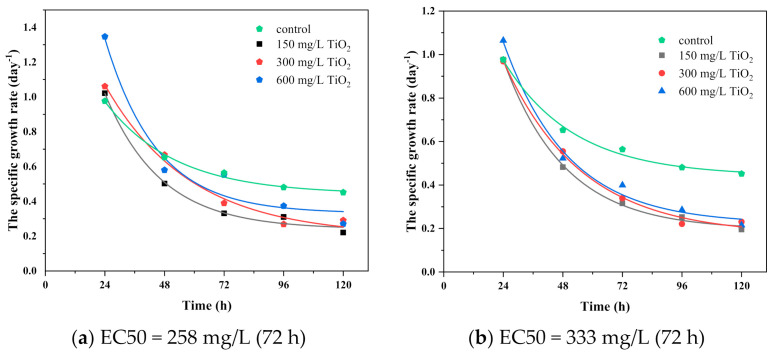

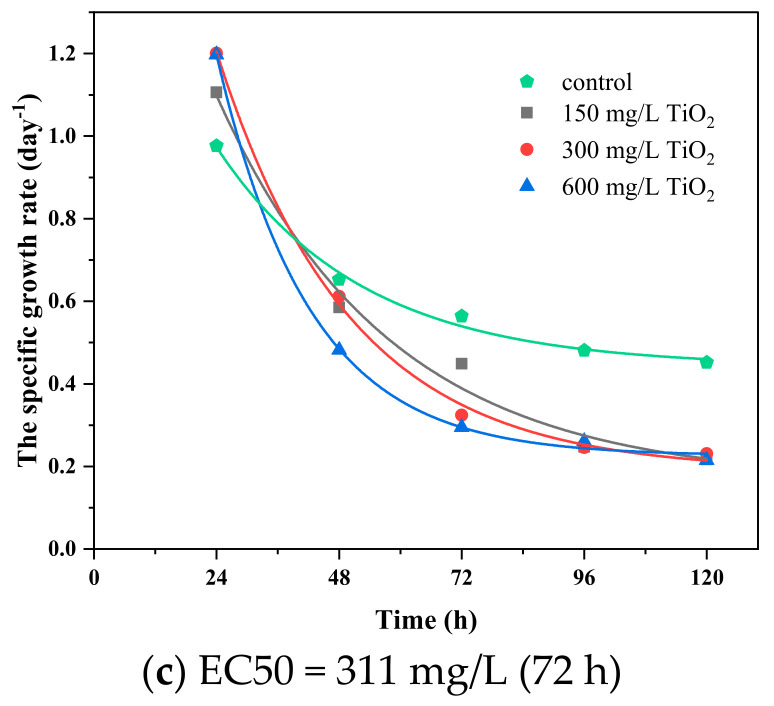
Growth curves of *Chlorella vulgaris* with different concentrations of TiO_2_ (150, 300, and 600 mg/L) when IMD had a constant value 5 mg/L (**a**), 10 mg/L (**b**), and 20 mg/L (**c**).

**Figure 9:**
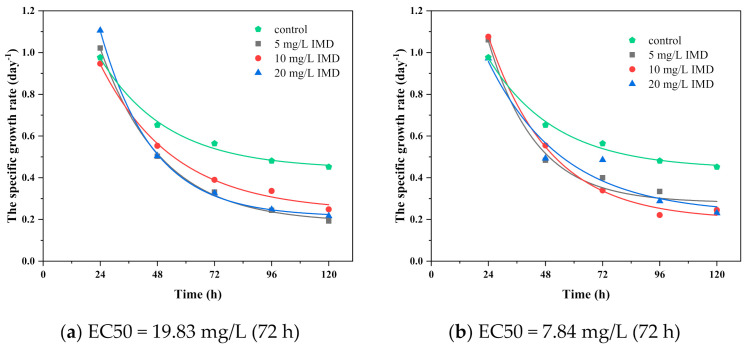

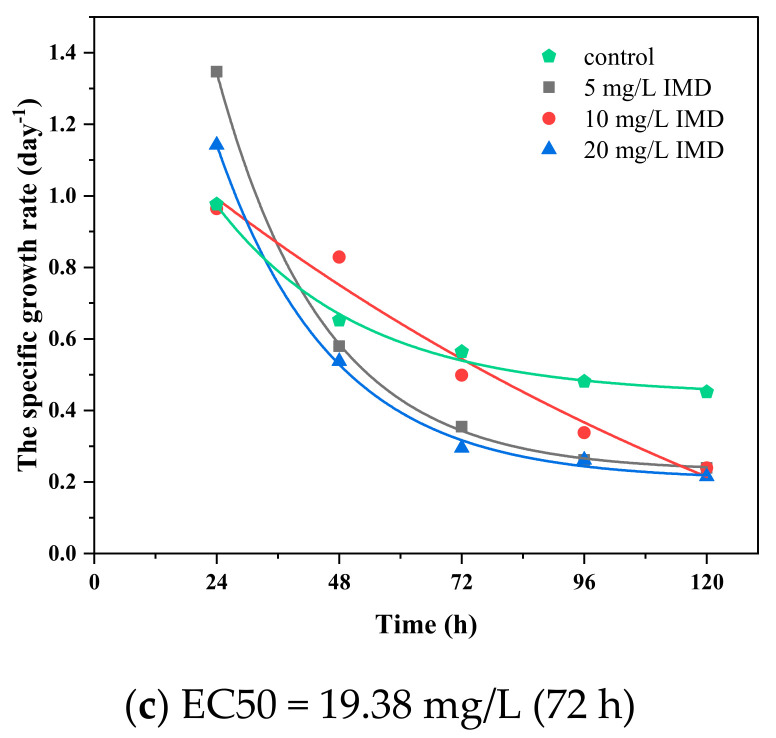
Growth curves of *Chlorella vulgaris* with different concentrations of IMD (5, 10, and 20 mg/L) when TiO_2_ had a constant value 150 mg/L (**a**), 300 mg/L (**b**), and 600 mg/L (**c**).

The inhibitory effect of IMD and TiO_2_ nanoparticles on *Chlorella vulgaris* is shown in Figure 8 and Figure 9. Figure 8 shows that the pesticide concentration remained constant. The concentration of TiO_2_ varied; therefore, the specific growth rate decreased in direct proportion to the increase in the concentration of IMD pesticide, which tended to stop the growth with increasing incubation time, underlining the exponential trend. The results indicate the development of *Chlorella vulgaris* inhibited by the IMD pesticide in the concentration of 5 mg/L with TiO_2_ nanoparticles in the concentration of 150, 300, and 600 mg/L (binary system toxicity) having an EC50 value after 72 h of 258.42 mg/L and an EC20 value after 72 h of 305.37 mg/L (Figure 8a). These values increased after 120 h, around 289.64 mg/L for EC50 and 342.26 mg/L for EC20, accentuating the toxic effect over time. In the case of IMD at a concentration of 10 mg/L, the EC50 value after 72 h was 333.78 mg/L and EC20 was 394.42 mg/L (Figure 8b). These values decreased after 120 h, around 317.47 mg/L for EC50 and 375.14 mg/L for EC20. In the IMD case at a concentration of 20 mg/L, the EC50 value after 72 h was 311.11 mg/L and EC20 was 367.63 mg/L (Figure 8c). These values decreased after 120 h, around 226.69 mg/L for EC50 and 191.84 mg/L for EC20. Imidacloprid is currently not actively regulated in marine ecosystems.

The presence of IMD and titanium dioxide strengthened the toxicity on *Chlorella vulgaris*, and the EC50 value decreased from 388 mg/L (in the absence of IMD) to 258 mg/L (when IMD was 5 mg/L).

Figure 9 shows that the concentration of TiO_2_ nanoparticles remained constant while the concentration of imidacloprid varied. As a result, the specific growth rate decreased in direct proportion to the increase in TiO_2_ concentration, which tended to stop the growth as the incubation time increased, highlighting the exponential trend. The results show that TiO_2_ suppressed the development of *Chlorella vulgaris* at 150 mg/L in combination with IMD at 5, 10, and 20 mg/L (binary system toxicity), with an EC50 of 19.83 mg/L after 72 h and an EC20 of 23.43 mg/L after 72 h (Figure 9a). After 120 h, these values dropped to roughly 8.95 mg/L for EC50 and 10.58 mg/L for EC20. After 72 h, the EC50 value for TiO_2_ at 300 mg/L was 7.84 mg/L, and the EC20 value was 9.26 mg/L (Figure 9b). After 120 h, these values remained rather constant, about 7.77 mg/L for EC50 and 9.18 mg/L for EC20. After 72 h, the EC50 value for TiO_2_ at 600 mg/L was 19.38 mg/L, and the EC20 value was 22.90 mg/L. (Figure 9c). After 120 h, these values dropped to roughly 10 mg/L for EC50 and 11.82 mg/L for EC20. The EC50 for IMD was approximately 19 mg/L regardless of the TiO_2_ concentration in the binary-toxic system, except for a TiO_2_ concentration of 300 mg/L, where IMD was 7.84 mg/L.

The precise causes of toxic effects are unknown, but various mechanisms could be responsible for nanomaterial and pesticide toxicity based on the species studied. For example, the toxicity of TiO_2_ may be due to its chemical properties (e.g., ion dissolution) or to the oxidative damage or conditions caused by the physicochemical properties of nanoparticles, which cause cellular component harm. The toxicity of this mixture increased as compared to the estimated value, and there was dependence on the concentration of the toxicant (the specific growth rate), meaning that the toxicity of the mixture can be calculated based on not only the toxicity of its constituents but also the relationship or a potential synergistic activity between TiO_2_ (Degussa) and imidacloprid.

The photodegradation process of imidacloprid (range of 5–40 mg/L) was also investigated in the algal medium incubated with 150–600 mg/L of titanium dioxide. During the 120-h of incubation of Degussa and IMD with algal cultures, samples were taken daily, and pesticide concentrations were analysed using HPLC. No degradation of IMD was found due to the photolysis mechanism. In contrast, the photodegradation efficiency of IMD increased from 28% to 38% when the IMD concentration decreased from 20 to 10 mg/L. Furthermore, a residual equilibrium concentration was observed after 96 h, 13 mg/L, and 8 mg/L, respectively, when the initial concentration was 20 and 10 mg/L. Further studies on how the photodegradation process influences the growth of algae will be performed.

### 3.5. The Dose–Response and Growth Kinetics

In most microbial toxicity assays, the response is a quantitative, continuous variable. For example, an increase in biomass could be used to quantify the specific growth rate, which is the rate of growth that is unique to an organism, environment, and culture medium. It could also be measured in terms of dry weight gain or loss, cell number, or pigment concentration [15,52]. However, in algal growth inhibition tests, the dose–response quantified as EC20 and EC50 was used.

The values of EC20 and EC50 for *Chlorella vulgaris* to various concentrations of TiO_2_, IMD, and their mixture are shown in Table 2. The results show that EC20 for TiO_2_ at a concentration of 150–2000 mg/L after 96 h was 567.72 mg/L, and EC50 was 428.39 mg/L. In fact, for IMD experiments, both values (EC20 and EC50) were smaller than those for TiO_2_, i.e., 16.21 and 11.76 mg/L. For the binary system toxicity, the smaller value for EC50 (9.05 mg/L) was obtained after 96 h with 600 mg/L of TiO_2_ and IMD at 5–20 mg/L.

Toxicity assays with algae as test organisms are a valuable tool for determining the biological effects of components in solution; the findings demonstrate that algae are susceptible to some hazardous compounds (Table 2). The degree of toxicity varies depending on the substance and the growth parameters used.

## 4. Conclusions

The organisms’ responses to different concentrations of TiO_2_-P25/20 and pesticide depend on particle type (e.g., size, surface area, shape) and exposure conditions (e.g., medium, pH, type of light, and intensity illumination). At low concentrations, titanium dioxide particles and imidacloprid have significant effects on the growth of the green microalga *Chlorella vulgaris*.

For the first time, this study demonstrated the cumulative effects of TiO_2_-P25/20 and imidacloprid (IMD) on the growth of the aquatic organism *Chlorella vulgaris*. The cumulative toxic effect of TiO_2_ and IMD presented a maximum at 96 h of exposure and decreased after 120 h due to the photocatalytic degradation of IMD. The effective concentration (EC50) experimentally determined after 120 h was 200–300 mg TiO_2_/L and 7–10 mg IMD/L, so the green alga is highly vulnerable to nanoparticles and pesticides. Thus, EC50 decreased for TiO_2_ from 290 to 220 mg/L and IMD from 12 to 7–10 mg/L in binary systems.

These findings point to a range of simulated/inhibitory responses. However, further research is needed to thoroughly understand the real-time and long-term cumulative effects of nanoparticles and pesticides in the environment. Furthermore, more in-depth studies are required to show the ecotoxicity of these pollutants more thoroughly and accurately, given their variety and complexity.

## Figures and Tables

**Table 1:** Toxicological characterisation of TiO_2_ exposure to different types of algae.

Species	Toxicological Effect		References
	EC50 (mg/L)	Time (h)	
*Chlorella vulgaris*	388	72	This study
	428	96	
	293	120	
*Karenia brevis*	10.69	72	[25]
*Skeletonema costatum*	7.37	72	[25]
*Raphidocelis subcapitata*	126.9		[38]
*Chlorella*	2.160 ± 0.06	72	[39]
*Scenedesmus*	4.139 ± 0.11	72	[39]
*Phaeodactylum tricornutum*	167.71	120	[35]
*Pseudokirchneriella subcapitata*	113	96	[40]
*Chlamydomonas reinhardtii*	>100	192	[41]
*Nitzschia closterium*	88.78	96	[42]

**Table 2:** The EC20 and EC50 for *Chlorella vulgaris* in terms of the single and binary response of TiO2 and IMD under 72, 96, and 120 h acute toxicity.

Toxicant	Concentration		72 h		96 h		120 h	
	TiO_2_	IMD	EC20	EC50	EC20	EC50	EC20	EC50
	mg/L	mg/L	mg/L	mg/L	mg/L	mg/L	mg/L	mg/L
TiO_2_	150…2000	-	529.67	388.14	567.72	428.39	1041.66	293.48
IMD	-	5…40	17.27	13.00	16.21	11.76	16.09	11.95
Binary system of TiO_2_ and IMD	150	5…20	23.43	19.83	11.82	10	10.58	8.95
	300	5…20	9.26	7.84	21.86	18.50	9.18	7.77
	600	5…20	22.90	19.38	10.69	9.05	11.82	10.00
	150…600	5	305.37	258.42	409.31	346.39	342.26	289.64
	150…600	10	394.42	333.78	270.52	228.93	375.14	317.47
	150…600	20	367.63	311.11	300.92	254.65	191.84	226.69

## Supplementary Materials

The following are available online at https://www.mdpi.com/article/10.3390/ijerph18157785/s1, Figure S1: Time depending results of biovolume from a batch culture of Chlorella Vulgaris under toxic binary TiO_2_ and IMD stress (IMD was kept constant at 5 mg/L), Figure S2: Time depending results of biovolume from a batch culture of Chlorella Vulgaris under toxic binary TiO_2_ and IMD stress (IMD was kept constant at 10 mg/L), Figure S3: Time depending results of biovolume from a batch culture of Chlorella Vulgaris under toxic binary TiO_2_ and IMD stress (IMD was kept constant at 20 mg/L), Table S1: Evaluation of physic-chemical parameters value of pH, conductivity and dissolved oxygen) on the effect of Chlorella vulgaris growth, Table S2: Evaluation of physico-chemical parameters (value of pH, conductivity and dissolved oxygen) on the effect of Chlorella vulgaris growth in the presence of 0, 150 … 2000 mg/L TiO_2_-P25/20), Table S3: Evaluation of physico-chemical parameters (value of pH, conductivity and dissolved oxygen) on the effect of Chlorella vulgaris growth in the presence of 0, 5 … 40 mg/L imidacloprid), Table S4: Evaluation of physico-chemical parameters (value of pH, conductivity and dissolved oxygen) on the effect of Chlorella vulgaris growth in the presence of mixture IMD and TiO_2_ NPs)
