# Supplementary material for: Toxicity of a Binary Mixture of TiO2 and Imidacloprid Applied to Chlorella vulgaris
Source: Int J Environ Res Public Health. 2021 Jul 22;18(15):7785. doi: 10.3390/ijerph18157785 (PMC8345346; doi:10.3390/ijerph18157785)
Supplement: Supplementary file 1 [file ijerph-18-07785-s001.zip › ijerph-1297299-supplementary.pdf]

Table S1. Evaluation of physic-chemical parameters value of pH, conductivity and dissolved oxygen) on the effect of Chlorella vulgaris growth

Day	pH	Conductivity (μS/cm)	Dissolved oxygen (ppm)
initial	8.1	1365	5.62
1	7.8	1363	5.63
2	8.05	1508	6.16
3	8.26	1465	5.74
4	9	1254	6.26
5	9.8	1441	6.94
6	9.8	1455	7.3
7	9.8	1334	7.94
8	9.96	1430	6.54
9	9.89	1470	7.65
10	9.88	1616	7.55
11	10.19	1448	7.01
12	10.2	1430	8.33
13	10.37	1443	8.37
14	10.61	1421	7.94

Table S2. Evaluation of physico-chemical parameters (value of pH, conductivity and dissolved oxygen) on the effect of Chlorella vulgaris growth in the presence of 0, 150 … 2000 mg/L TiO_2-P25/20)

Sample (mg/L)	Initial parameters (0 h)			Final parameters (after 120 h)		
	pH	Conductivity (μS/cm)	Dissolved oxygen (ppm)	pH	Conductivity (μS/cm)	Dissolved oxygen (ppm)
control	8.1	1341	7.8	9	1367	7.95
150	8.1	1339	7.67	8.9	1342	7.83
300	8.1	1339	7.43	8.7	1345	7.65
600	8.1	1337	7.71	8.9	1347	7.9
1200	8.1	1328	7.56	8.8	1349	7.77
2000	8.1	1324	7.49	8.8	1345	7.64

Table S3. Evaluation of physico-chemical parameters (value of pH, conductivity and dissolved oxygen) on the effect of Chlorella vulgaris growth in the presence of 0, 5 … 40 mg/L imidacloprid)

Sample (mg/L)	Initial parameters (0 h)			Final parameters (after 120 h)		
	pH	Conductivity (μS/cm)	Dissolved oxygen (ppm)	pH	Conductivity (μS/cm)	Dissolved oxygen (ppm)
control	8.1	1341	7.8	9	1367	7.95
5	8.1	1335	7.26	8.9	1342	7.43
10	8.1	1328	7.51	8.7	1341	7.61
20	8.1	1330	7.7	8.8	1341	7.89
30	8.1	1333	7.3	8.7	1342	7.54

40	8.1	1326	7.16	8.6	1346	7.34

Table S4. Evaluation of physico-chemical parameters (value of pH, conductivity and dissolved oxygen) on the effect of Chlorella vulgaris growth in the presence of mixture IMD and TiO_2 NPs)

IMD (mg/L)	TiO_2 (mg/L)	Initial parameters (0 h)			Final parameters (after 120 h)		
		pH	Conductivity (µS/cm)	Dissolved oxygen (ppm)	pH	Conductivity (µS/cm)	Dissolved oxygen (ppm)
0	0	8.1	1341	7.8	9	1367	7.95
	150	8.1	1337	7.67	8.82	1348	7.71
5	300	8.1	1341	7.72	8.76	1354	7.77
	600	8.1	1346	7.64	8.89	1359	7.78
	150	8.1	1340	7.83	8.69	1353	7.89
10	300	8.1	1342	7.81	8.74	1359	7.9
	600	8.1	1344	7.79	8.85	1368	7.86
	150	8.1	1346	7.87	8.9	1352	7.9
20	300	8.1	1348	7.84	8.97	1364	7.9
	600	8.1	1351	7.86	9	1370	7.89

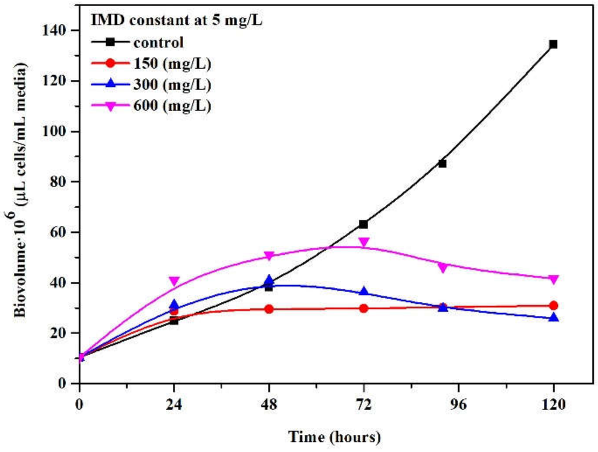

Figure S1. Time depending results of biovolume from a batch culture of Chlorella Vulgaris under toxic binary TiO_2 and IMD stress (IMD was kept constant at 5 mg/L).

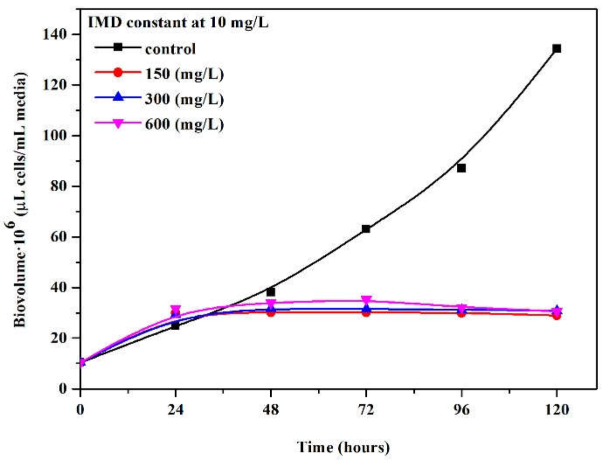

Figure S2. Time depending results of biovolume from a batch culture of Chlorella Vulgaris under toxic binary TiO_2 and IMD stress (IMD was kept constant at 10 mg/L).

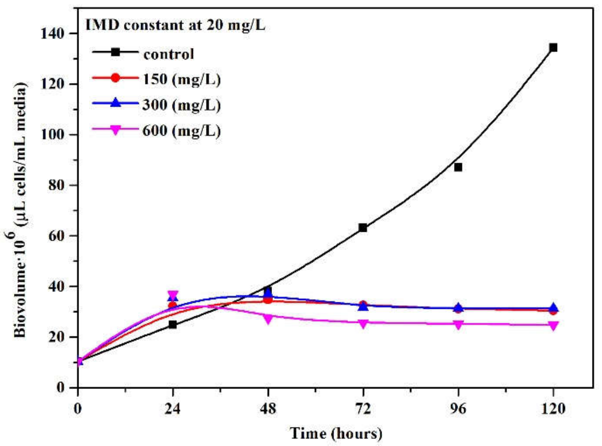

Figure S3. Time depending results of biovolume from a batch culture of Chlorella Vulgaris under toxic binary TiO_2 and IMD stress (IMD was kept constant at 20 mg/L).
